# Inhibition of poly-LacNAc biosynthesis with release of CMP-Neu5Ac feedback inhibition increases the sialylation of recombinant EPO produced in CHO cells

**DOI:** 10.1038/s41598-018-25580-9

**Published:** 2018-05-08

**Authors:** Chung-Geun Lee, Myung Jin Oh, Seung-Yeol Park, Hyun Joo An, Jung Hoe Kim

**Affiliations:** 1 Department of Biological Sciences, Korea Advanced Institute of Science and Technology, 291 Daehak-ro, Yuseong-gu, Daejeon, 34141 Republic of Korea; 2 Graduate School of Analytical Science & Technology, Chungnam National University, 99 Daehak-ro, Yuseong-gu, Daejeon, 34134 Republic of Korea; 3 Asia-pacific Glycomics Reference Site, Daejeon, 34134 Republic of Korea; 4 Division of Rheumatology, Immunology and Allergy, Brigham and Women’s Hospital, and Department of Medicine, Harvard Medical School, Boston, MA 02115 USA

## Abstract

Sialylation of recombinant therapeutic glycoproteins modulates their pharmacokinetic properties by affecting their *in vivo* half-life. N-glycan branching on glycoproteins increases the number of potential attachment sites for sialic acid. Here, we introduce a new approach for increasing the sialylation of recombinant human erythropoietin (rhEPO) produced in CHO cells by modulating poly-N-acetyllactosamine (poly-LacNAc) biosynthesis. We did not observe an increase in rhEPO sialylation, however, until the feedback inhibition by intracellular cytidine monophosphate-N-acetylneuraminic acid (CMP-Neu5Ac), which is a limiting factor for sialylation, was released. Thus, we found that a combined approach inhibiting poly-LacNAc biosynthesis and releasing CMP-Neu5Ac feedback inhibition produces the most significant increase in rhEPO sialylation in metabolically engineered CHO cells. Furthermore, a detailed analysis of the resulting N-glycan structures using LC/MS revealed increased tri- and tetra- sialylated N-glycan structures accompanied by a reduction of di-sialylated N-glycan structures. These results validate our new approach for glycosylation engineering, and we expect this approach will be useful in future efforts to enhance the efficacy of other therapeutic glycoproteins.

## Introduction

Glycosylation contributes to several properties of therapeutic drugs, such as pharmacokinetics, and immunogenicity^1,2^. Proper modulation of glycosylation is a major issue in producing recombinant therapeutic glycoproteins^3,4^. Sialylation of a glycoprotein’s N-glycans modulates the life span over which that specific protein molecule circulates *in vivo*. This occurs because sialylation prevents the recognition of galactosyl residues by hepatocyte asialoglycoprotein receptors (ASGPRs), which triggers protein degradation^5,6^. Thus, various methods have been developed for increasing sialylation^7–13^.

N-acetylglucosamine (GlcNAc) is the initial glycan residue in N-glycan branches. Initial N-glycan branches are extended by the sequential attachment of galactosyl- and sialyl- residues. The degree of N-glycan branching determines the number of available sites for the attachment of terminal sialic acid residues. Early studies found that antennary structures are related to the intracellular level of UDP-GlcNAc^14^. GlcNAc supplementation during cell culture increases N-glycan branching^15^. Subsequently, the storage of UDP-GlcNAc was found to be limited such that excess UDP-GlcNAc is shunted toward other glycosylation pathways as well^16^. Another attempt at enhancing N-glycan branching involved the overexpression of α-1,3-mannosyl-glycoprotein 2-β-N-acetylglucosaminyltransferase (MGAT), which is required to form the GlcNAc branching on the N-glycan core structure (Man3GlcNAc2). Notably, while overexpression of MGAT-4 and MGAT-5 does induce N-glycan branching, it also increases poly-LacNAc chain formation^17,18^.

**Figure 1:**
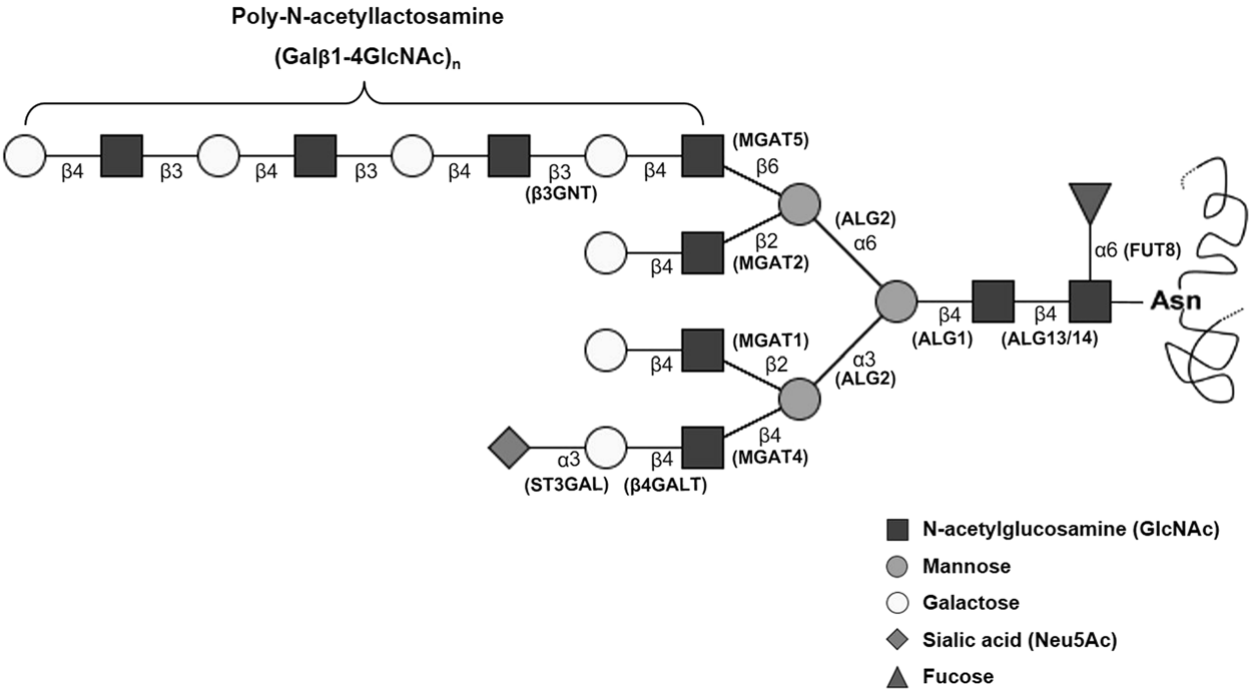
Schematic structure of a typical poly-LacNAc type N-glycan. Poly-N-acetyllactosamine (Poly-LacNAc) comprises repeated Galβ1-4GlcNAc disaccharides, called N-acetyllactosamine (LacNAc). The biosynthesis of poly-LacNAc is accomplished by the enzymatic action of β-1,3 N-acetylglucosaminyltransferase (β3gnt) which catalyzes the addition of GlcNAc to N-glycan Gal termini. ALG, asparagine linked glycosylation gene, FUT, fucosyltransferase, MGAT, mannosyl-glycoprotein N-acetylglucosaminyltransferase, β4GALT, beta-1,4 galactosyltransferase, ST3GAL, beta-1,4-galactoside alpha-2,3 sialyltranferase, β3GNT, beta-1,3 N-acetylglucosaminyltransferase.

Poly-LacNAc consists of repeated N-acetyllactosamine (Galβ1-4GlcNAc)_n_ residues formed as GlcNAc residues are attached to galactosyl termini via the enzymatic activity of β-1,3 N-acetylglucosaminyltransferase (β3gnt) (Fig. 1). Of the several homologs of β3gnt, β3gnt2 is crucial enzyme for synthesis of poly-LacNAc in CHO cells^19^. rhEPO containing poly-LacNAc is cleared from the blood faster than the EPO lacking poly-LacNAc^5^. Thus, reducing poly-LacNAc chains on therapeutic recombinant glycoproteins improves their half-life and efficacy *in vivo*. The biosynthetic pathways that form N-glycan branches and poly-LacNAc chains both utilize GlcNAc and galactose as building blocks. Thus, we hypothesized that rhEPO sialylation may increase when poly-LacNAc synthesis is inhibited because this would increase the availability of GlcNAc and galactosyl residues for N-glycan branch synthesis.

In this study, we introduce a new approach for increasing rhEPO sialylation by modulating N-glycan branch synthesis. We found that β3gnt2-depleted CHO cells produce rhEPO without poly-LacNAc, but with increased tri- and tetra-antennary N-glycans. Notably, however, sialylation of rhEPO from β3gnt2-depleted CHO cells were still not increased due to feedback regulation by intracellular CMP-Neu5Ac^20,21^. We were able to overcome this issue by depleting β3gnt2 in EC2-1H9-CTSTrEKm cells, which are insensitive to this feedback inhibition. In summary, by modifying CHO cells to increase N-glycan branching synthesis and reduce feedback inhibition by CMP-Neu5Ac, we were able to produce rhEPO with significantly more N-glycan branching and sialylation.

## Results

### Knock-down of β3gnt2 reduces polylactosaminylation of rhEPO

**Figure 2:**
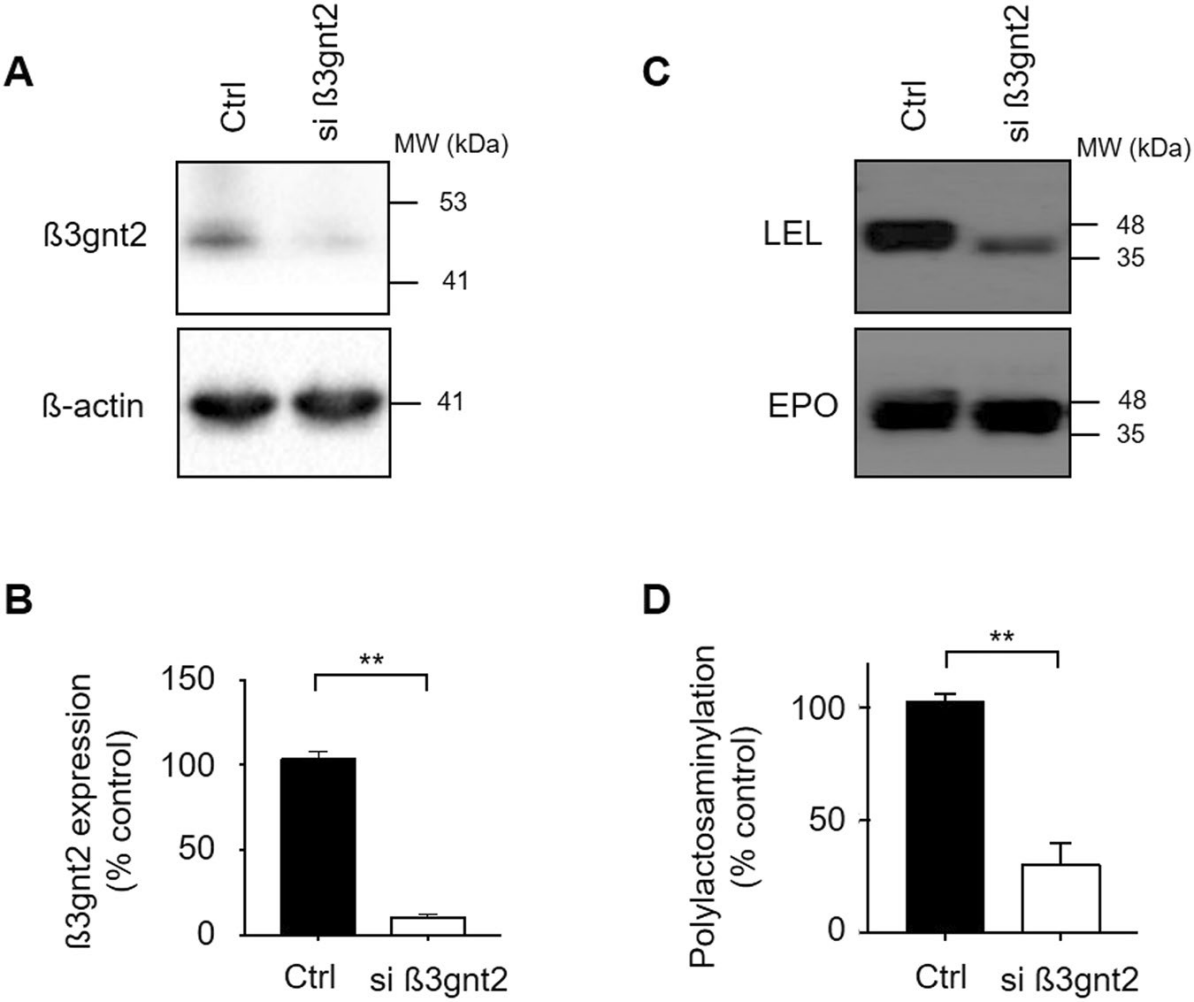
Decreased poly-LacNAc via *β3gnt2* knock-down. (**A**) Depletion of β3gnt2 by siRNA was determined by western blot. Lane 1, EC2-1H9 cells. Lane 2, EC2-1H9 cells treated with siRNA against β3gnt2. (**B**) Quantification of panel A using Image J. The band intensities of β3gnt2 were normalized by the intensities of β-actin. (**C**) Lectin blot results show reduced polylactosaminylation of rhEPO purified from β3gnt2*-*depleted CHO cells. For detection of polylactosaminylation, 1 μg of purified rhEPO was used. (**D**) Quantification of the data in C. LEL lectin band intensities were normalized by EPO band intensities. The data shown are presented as means ± S.E.M. from three independent experiments each with duplicate samples. **P < 0.01 (Student’s t-test).

We first attempted to reduce poly-LacNAc by inhibiting the expression of β3gnt2 using siRNA. We expected this would increase N-glycan branching and terminal sialylation. To this end, we treated the rhEPO-producing CHO cell line EC2-1H9 with siRNAs designed by Invitrogen to specifically target the *β3gnt2* sequence listed in GenBank (NCBI). Three days after siRNA transfection, we harvested the cells and analyzed the expression of β3gnt2 by western blot. As shown in Fig. 2A and B, siRNA-mediated knockdown effectively reduces β3gnt2 expression in the transfected cells. We next validated the effect of *β3gnt2* knock-down with poly-LacNAc-specific lectin. After pulling down rhEPO from cell supernatants using an anti-EPO antibody, we performed a western blot using Lycopersicon esculentum lectin (LEL) and the same anti-EPO antibody. Consistent with the expression pattern of β3gnt2, rhEPO from β3gnt2*-*depleted CHO cells showed a reduced level of poly-LacNAc when compared to control cells (Fig. 2C and D).

### Inhibition of polyLacNAc biosynthesis enhances N-glycan branching

**Figure 3:**
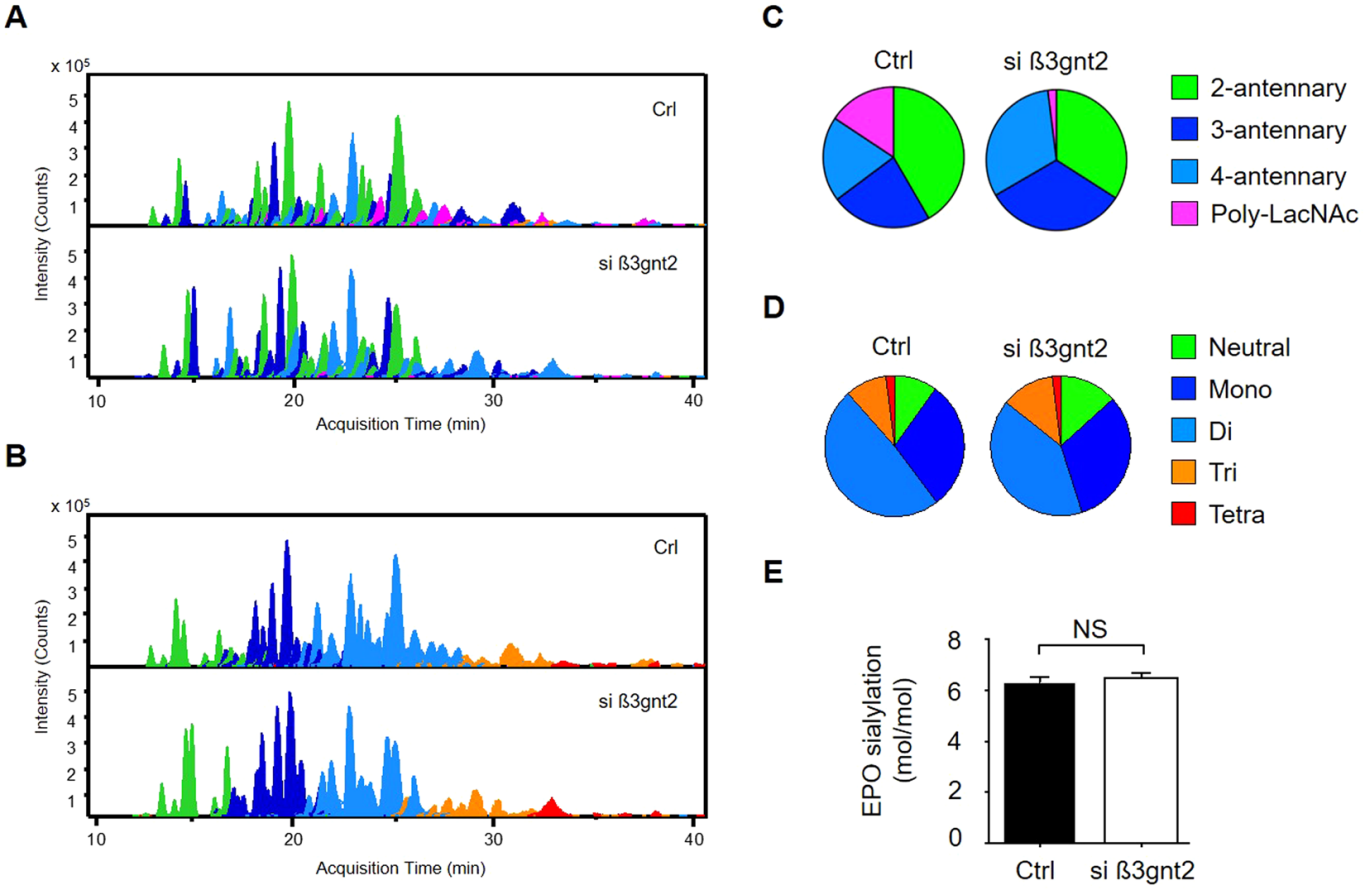
Comparison of N-glycan profiles and sialic acid contents. Distributions of N-glycan antennal branching (**A**) and sialylation (**B**) presented using Extracted Compound Chromatograms (ECCs) with the control above and the β3gnt2-depleted cells below. The data in (**A**) and (**B**) are quantified in (**C**) and (**D**), respectively. (**E**) Sialic acid content was determined using the OPD-labeling method. The data shown are presented as means ±S.E.M. from three independent experiments each with duplicate samples. NS, not significant (Student’s t-test).

Through western blot experiments, we found that knock-down of *β3gnt2* decreases the polylactosaminylation of rhEPO. In order to confirm our observation, released N-glycans on rhEPO were analyzed using a chip-based nano-LC/qTOF-MS system. Representative extracted compound chromatograms (ECCs) of N-glycans identified in rhEPO were shown in Fig. S1. Initially, N-glycan compositions were assigned according their accurate masses and known EPO glycosylation^22^. Modifications such as O-acetylation (OAc) and dehydration (-H_2_O) often found in EPOs was also considered^23–25^. Total thirty two N-glycan compositions corresponding to 62 isomer-specific molecules were separated on a PGC column. In general, smaller and neutral glycans eluted earlier, while larger and acidic glycans eluted later. Glycan structures were further identified by the combination of MS/MS analysis and glyco-synthetic pathway. The intensity of each peak was normalized across total glycan intensities to determine the distribution of N-glycan branching (Fig. 3A and C) and the degree of sialylation (Fig. 3B and D), respectively. The classification of glycan branching could be basically determined by the number of N-acetylhexosamine (HexNAc) in N-glycan composition considering N-glycan biosynthesis process. For example, we categorized N-glycans having four HexNAc into bi-antennary group because two HexNcA attached to asparagine on a protein, and leaving two HexNAc attached on N-glycan core as two antennae. Although polylactosaminylated bi- or tri-antennary N-glycans are biologically possible, most polylactosaminylation on recombinant EPOs was found in tetra-antennary N-glycans^24,26,27^. Indeed, polylactosaminylation was observed to have primarily on tetra-antennary rather than bi- or tri-antennary N-glycans by our previous study^22^. Consistent with our lectin blot results, we found a drastic reduction in the ratio of polyLacNAc from 15.68% to 1.99%. Moreover, rhEPO produced in siRNA-treated CHO cells showed increased tri-antennary and tetra-antennary N-glycans when compared with rhEPO produced in control CHO cells (32.68% vs. 23.13% for tri-antennary N-glycans; 31.44% vs. 19.54% for tetra-antennary N-glycans). Bi-antennary N-glycans, in contrast, were reduced from 41.67% to 34.12%. In terms of sialylation itself, however, we did not observe any significant difference in the distribution of sialylated N-glycans (Fig. 3B and D) or in the amount of total sialic acid content (Fig. 3E) despite the increase in antennal branching. We supposed this unaltered sialylation was due to shortage of intracellular CMP-Neu5Ac pool in the cells. To identify this hypothesis, we measured intracellular CMP-Neu5Ac as described in methods section. We found that the intracellular CMP-Neu5Ac levels in *β3gnt2*-specific siRNA-treated EC2-1H9 cells were similar to control (Fig. S2A). Next, we measured the expression level of *st3gal4*, which encodes a major α2,3-sialyltransferase^28,29^, in both control CHO cells and siRNA-treated EC2-1H9 CHO cells. We found no significant change in the level of *st3gal4* caused by β3gnt2 depletion (Fig. S2B). Furthermore, in our detailed analysis, we found that the increased tri- and tetra-antennary N-glycans tend to have terminal galactose residues uncapped by sialic acid residues (Table S1). This suggests intracellular CMP-Neu5Ac may be the limiting factor reducing N-glycan branching and sialylation rather than galactose.

### Increased N-glycan branching further enhances sialylation of rhEPO

**Figure 4:**
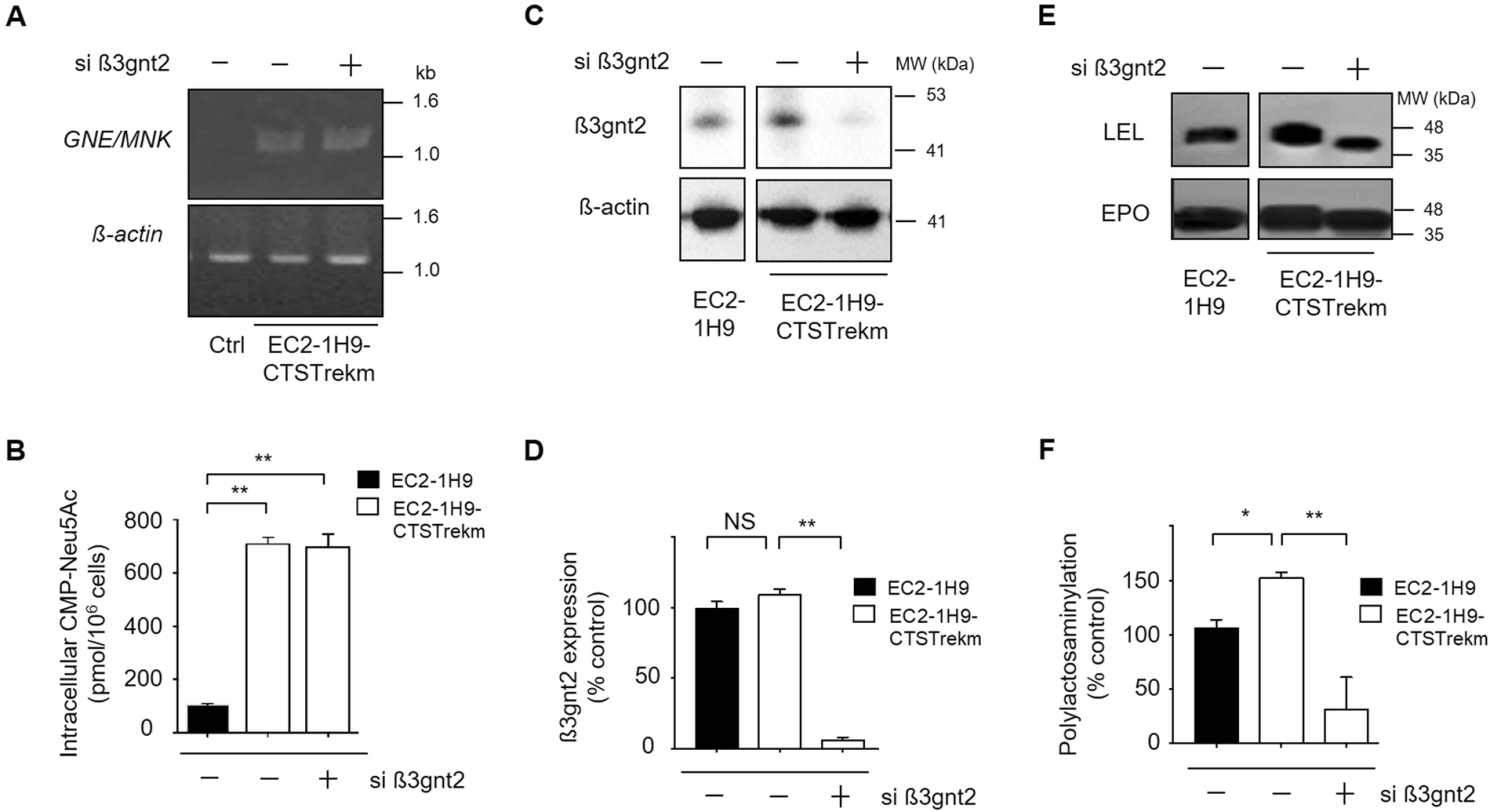
Poly-LacNAc is reduced in CHO cells with high intracellular CMP-Neu5Ac. (**A**) The over-expression of *GNE* in EC2-1H9-CTSTrekm9 confirmed by RT-PCR. Lane 1, EC2-1H9 cells; Lane 2, EC2-1H9-CTSTrekm cells; Lane 3, EC2-1H9-CTSTrekm cells treated with *β3gnt2*-specific siRNAs. (**B**) Bypassing CMP-Neu5Ac feedback inhibition via *GNE* over-expression increases intracellular CMP-Neu5Ac. (**C**) Expression of β3gnt2 was examined in EC2-1H9-CTSTrekm cells with depletion of β3gnt2 using siRNA. Lane 1, EC2-1H9 cells. Lane 2, EC2-1H9-CTSTrekm cells. Lane 3, EC2-1H9-CTSTrekm cells treated with siRNA against β3gnt2. (**D**) Quantification of panel C was shown. The band intensities of β3gnt2 were normalized by the intensities of β-actin. (**E**) Polylactosaminylation of purified rhEPO measured by western blot using LEL. For detection of polylactosaminylation, 1 μg of purified rhEPO was used. (**F**) Quantification of the data in C. The LEL lectin band intensity was normalized by the EPO band intensity. The data shown are presented as means ± S.E.M. from three independent experiments each with duplicate samples. **P < 0.01, *P < 0.05 (Student’s t-test).

Under the assumption that insufficient intracellular pools of CMP-Neu5Ac are limiting N-glycan branching and sialylation in the context of reduced polyLacNAc synthesis, we attempted to regulate polylactosaminylation in EC2-1H9-CTSTrekm cells. EC2-1H9-CTSTrekm are CHO cells engineered to show increased sialylation via the over-expression of sialuria-like mutated rat UDP-N-acetyl-glucosamine-2-epimerase (GNE), CMP-Neu5Ac transporter (CMP-SAT), and human α2,3-sialyltransferase (ST)^30^. As in previous experiments, we transfected EC2-1H9-CTSTrekm cells with *β3gnt2*-specific siRNAs. After confirming their over-expression of *GNE* by RT-PCR (Fig. 4A), we measured intracellular CMP-Neu5Ac of EC2-1H9, EC2-1H9-CTSTrekm and β3gnt2-depleted EC2-1H9-CTSTrekm. We found the increased expression of GNE in EC2-1H9-CTSTrekm led to a 7-fold increase of intracellular CMP-Neu5Ac when compared to EC2-1H9 control cells (Fig. 4B). We also confirmed the siRNA-mediated depletion of β3gnt2 by western blot (Fig. 4C and D). Before determining whether this increased intracellular sialic acid led to further sialylation of the expanded N-glycan branches, we first performed a western blot experiment using LEL and confirmed a reduced level of polyLacNAc induced by *β3gnt2* knock-down (Fig. 4E and F).

**Figure 5:**
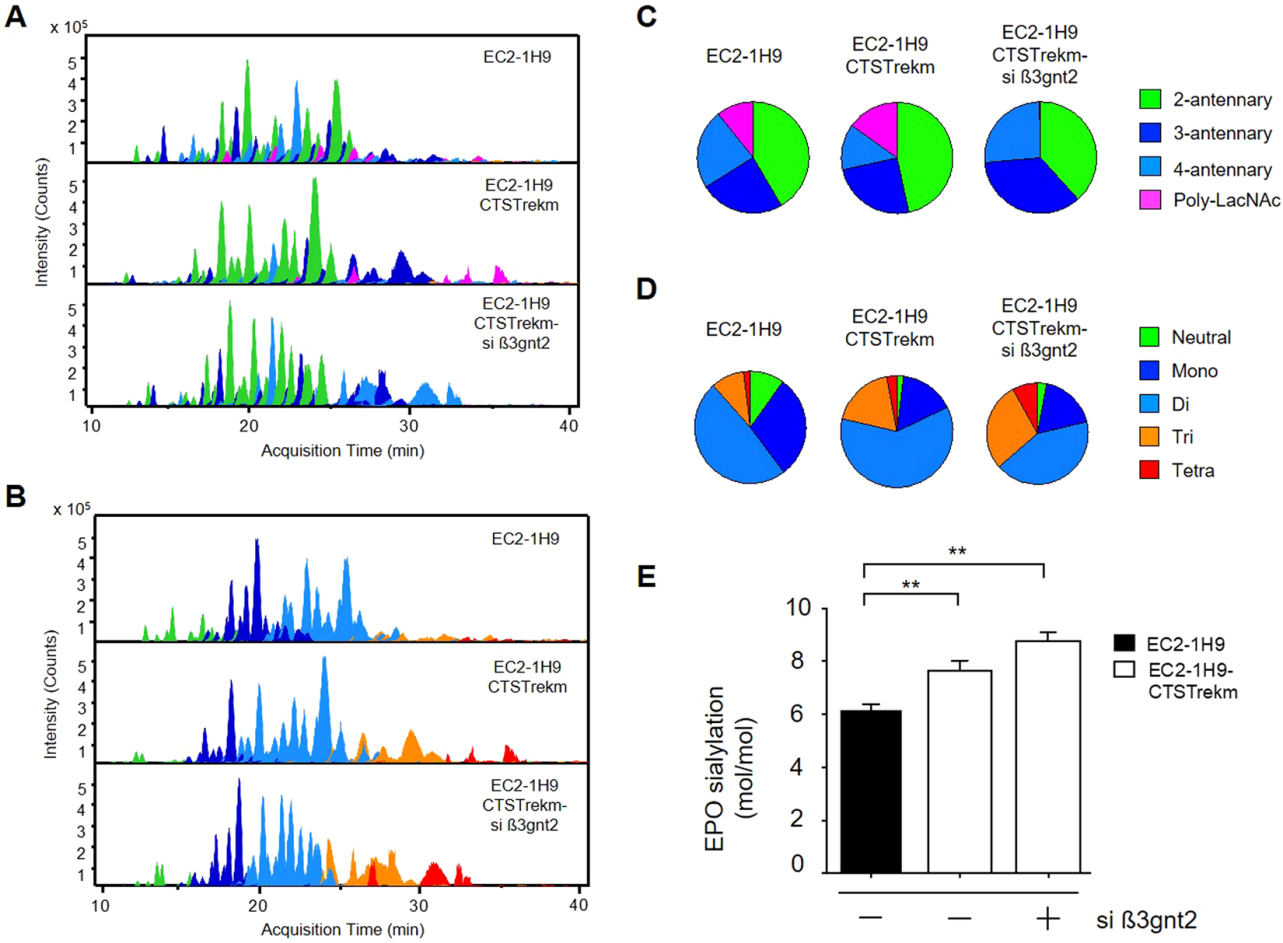
Enhanced N-glycan branch formation leads to a further increase in rhEPO sialylation. N-glycan antennary distribution (**A**) and sialylation (**B**) presented using Extracted Compound Chromatograms (ECCs) with EC2-1H9 control above, EC2-1H9-CTSTrekm in the middle, and β3gnt2-depleted EC2-1H9-CTSTrekm below. The data in (**A**) and (**B**) are quantified in (**C**) and (**D**), respectively. (**E**) Sialic acid content was determined using the OPD-labeling method. The data shown are presented as means ±S.E.M. from three independent experiments each with duplicate samples. **P < 0.01 (Student’s t-test).

We next performed an LC/MS experiment to identify qualitative and quantitative changes of N-glycan antennary structures and the degree of terminal sialylation following reduced poly-LacNAc by β3gnt2 inhibition. As shown in Fig. 5A to D, we found altered sialylation patterns associated with changes in N-glycan antennary distributions. In particular, tri-sialylated N-glycans were significantly increased to 28.30% in rhEPO produced from EC2-1H9-CTSTrekm cells treated with *β3gnt2*-specific siRNAs compared with rhEPO from control EC2-1H9-CTSTrekm cells (18.36%). The level of tetra-sialylated N-glycans was also elevated from 2.93% in control cells to 8.05% in β3gnt2-depleted EC2-1H9-CTSTrekm cells, despite the total ratio remaining less than 10%. Finally, we measured rhEPO sialylation via derivatization of sialic acids with OPD as described in the Methods section (Fig. 5E). Human erythropoietin has a maximum sialic acid content of 14 moles because it has 3 N-glycan sites capable of accepting 4 sialic acid residues each and 1 O-glycan site capable of accepting 2 sialic acid residues. While EC2-1H9-CTSTrekm and EC2-1H9 cells produce rhEPO with 7.6 moles and 6.2 moles of sialic acid, respectively, we found an average of 8.8 moles of sialic acid associated with the rhEPO produced by β3gnt2-depleted EC2-1H9-CTSTrekm cells.

## Discussion

Many attempts have been made to promote the sialylation of therapeutic proteins in an effort to enhance their stability *in vivo*. These include attempts at N-acetylmannosamine (ManNAc) supplementation^8^ and CMP-Neu5Ac transporter overexpression^9,10^. Recently, we reported enhanced sialylation of rhEPO by modulation of intracellular CMP-Neu5Ac, which is a key limiting factor for sialylation^11^.

Here, we further improve rhEPO sialylation by not only modulating intracellular CMP-Neu5Ac, but by additionally enhancing N-glycan branch formation. In previous studies, recombinant interferon (IFN)**-**γ and rhEPO with more antennary structures were obtained via the overexpression of MGAT-4 and −5, which are responsible for the formation of tri- and tetra-antennary structures^17,18^. These studies, however, found that this increased N-glycan branch formation was accompanied by increases in poly-LacNAc, which itself reduces protein stability *in vivo*. We noted several advantages of restraining the polylactosaminylation of glycoproteins. First, the inhibition of poly-LacNAc biosynthesis can increase N-glycan branch formation by shunting GlcNAc and galactose from linear elongation (poly-LacNAc) to horizontal extension (branching)^14–16^. Second, the *in vivo* half-life of rhEPO with poly-LacNAc was much shorter than rhEPO without poly-LacNAc^5^. Consistent with previous studies^14–16^, we found that inhibition of poly-LacNAc synthesis induces the formation of N-glycans with more antennary structures. We were therefore surprised to find that rhEPO produced in β3gnt2-depleted CHO cells did not show significantly more sialylation than that produced in control cells.

Intracellular CMP-Neu5Ac was found to limit sialylation^20,21^. We therefore suspected feedback inhibition induced by the accumulation of intracellular CMP-Neu5Ac was limiting the sialylation of rhEPO produced by β3gnt2-depleted CHO cells. Previously, we were able to overcome this issue via the overexpression of a mutated rat GNE^30^. We therefore sought to further increase rhEPO sialylation using EC2-1H9-CTSTrEKm CHO cells, which overexpress key sialylation enzymes. By knocking down *β3gnt2* in these EC2-1H9-CTSTrEKm cells, we were able to obtain rhEPO with both increased sialylation and improved N-glycan branching. A detailed analysis of N-glycosylation for this rhEPO using LC/MS revealed increases in tri- and tetra-sialylated N-glycans of more than 1.5- and 2.7-fold, respectively. We found, though, that these tri- and tetra- antennary structures were not fully occupied by sialic acid residues. It is possible that secreted sialidases are hydrolyzing the terminal sialyl residues of rhEPO^31–33^. This hypothesis should be addressed in future studies.

In summary, we present here a comprehensive approach for enhancing the sialylation of rhEPO produced from CHO cells by modulating poly-LacNAc synthesis. Inhibiting poly-LacNAc synthesis prevents the inefficient use of GlcNAc and galactose residues, shunting them into the formation of more antennary structures, which allow for enhanced sialylation. The further release of feedback inhibition by intracellular CMP-Neu5Ac promotes sialylation of the increased antennary structures. This new approach represents a significant contribution to the optimized therapeutic efficacy of not only rhEPO, but also other recombinant therapeutic glycoproteins.

## Materials and Methods

### Cell lines and culture maintenance

EC2-1H9, a CHO cell line producing recombinant human EPO, was provided by Dr. Hyo Jeong Hong (Antibody Engineering Research Unit, Korea Research Institute of Bioscience and Biotechnology, Yuseong-gu, Daejeon, Korea). EC2-1H9-CTSTrekm is a sialylation pathway enhanced cell line previously constructed in our group based on EC2-1H9. This cell line was constructed by overexpressing mutated rat UDP-N-acetyl-glucosamine-2-epimerase (GNE), CMP-Neu5Ac transporter (CMP-SAT) and human α2,3-sialyltransferase (ST). Cell lines were cultured with MEM-α (Gibco) supplemented with 10% (v/v) FBS (Gibco), 3.5 g/L glucose, 20 nM MTX (methotrexate; Sigma), and 1% (v/v) Ab-Am (antibiotic-antimycotic solution; Gibco) in a humidified atmosphere containing 5% CO_2_ at 37 °C. For the EC2-1H9-CTSTrekm cells, 400 μg/mL hygromycin B (Invitrogen) and 500 μg/mL Zeocin (Invitrogen) were added for the selection of the transfected genes.

### Inhibition of β3gnt2 expression by siRNA transfection

The siRNA sequence targeting Chinese hamster *β3gnt2* mRNA (GenBank accession no. XM_003502146.3) was obtained and annealed by Invitrogen (Carlsbad, CA); sense strand: 5′-GAAGAAATGCGCAAAGAA-3′, anti-sense strand: 5′TTCTTTGCGCATTTCTTC-3′. The siRNA was transfected into CHO cells using Lipofectamine™ RNAiMAX reagent (Invitrogen, Carlsbad, CA) according to the manufacturer’s instructions. Twenty-four hours after siRNA transfection, the culture media was replaced with serum-free media (CHO-S-SFM II; Gibco) to enrich for rhEPO and exclude serum proteins. Three days after this media change, the cells were harvested to analyze the effects of siRNA-mediated mRNA suppression.

### Determination of expression level of genes by RT-PCR and qRT-PCR

Total RNA was extracted from transfected CHO cells using RNeasy Mini Kit from Qiagen. One μg of this total RNA isolated from each cell type was used as a template for cDNA production via RT-PCR using the AccuPower RT-PCR PreMix (Bioneer) according to the manufacturer’s instructions. *GNE/MNK* transcript levels were determined by RT-PCR using following primer pair: forward, 5′- GGT GAC CAC CGA CAT TAA GCA TTC C-3′; reverse, 5′- GGA CAG GAG AAC TTT GTG ACG CTC-3. *β-actin* was simultaneously amplified as an internal control using 5′-ATG GAT GAC GAT ATC GCT GCG CTC G-3′ and 5′-CCA TCG TCC ACC GCA AAT GCT TCT AG-3′ for the forward and reverse primers, respectively. The amplified PCR products were separated on a 0.8% agarose gel and visualized by ethidium bromide (EtBr) staining. The band intensity of *GNE/MNK* was normalized by intensity of *β-actin*.

The expression of endogenous *st3gal4* was measured by qRT-PCR using a CFX96 touch real-Time PCR detection system (Bio-Rad, Hercules, CA) and iQ^TM^ SYBR Green Supermix (Bio-Rad, Hercules, CA) according to the manufacturer’s instructions. *β-actin* was simultaneously amplified as an internal control. The primers used were: *st3gal4* forward, 5′-AGT GTC GTC GTT GTG TTG TG-3′; reverse, 5′-AGA AAA CAA CCC AGA CAC GC-3′; *β-actin* forward, 5′-AGC TGA GAG GGA AAT TGT GCG-3′; reverse 5′-AAT GAG CGG TTC CGT TGC-3′.

### Western blotting

β3gnt2 expression was measured by western blotting. Cells were lysed using RIPA buffer (Thermo Scientific; Rockford, IL) according to the manufacturer’s instructions. After harvesting total protein extracts from each cell sample, their concentration was measured using the Quant-iT™ protein assay kit (Invitrogen). A 10 μg sample of each total cell lysate was subjected to SDS-PAGE and transferred to a PVDF membrane (Millipore Corp., Bedford, MA). These membranes were blocked with 5% BSA in TBS-T (140 mM NaCl, 10 mM Tris-HCl, and 0.05% Tween 20, pH 8.0) for an hour at room temperature followed by a 4 °C overnight incubation with biotinylated antibodies for β3gnt2 and β-actin. Then, the membranes were washed three times for 5 min with TBS-T and incubated at room temperature for 1 hour with an HRP-conjugated mouse-anti-goat IgG antibody for β3gnt2 and a goat-anti-mouse IgG antibody (Santa Cruz) to detect the β-actin antibody. For detection of polylactosaminylation, 1 μg of purified rhEPO and biotinylated antibodies (*Lycopersicon Esculentum* Lectin; LEL, anti-EPO antibody) were used. After incubation with the antibodies, ExtrAvidin-peroxidase (Sigma) for LEL and HRP-conjugated goat-anti-mouse IgG antibody (Santa Cruz) for the anti-EPO antibody were used as secondary antibodies. The blots were then developed using an ECL kit (Thermo Scientific; Rockford, IL). Signal quantification was performed using the ImageJ software from the NIH. β3gnt2 and LEL band intensities were normalized by the signal from the β-actin antibody and anti-EPO antibody, respectively.

### Purification of rhEPO and determination of its sialic acid contents

Three days after replacing the media with serum-free media, the collected culture media was filtered using 0.45 μm filters (Sartorius, Göttingen, Germany) and dialyzed against PBS (pH 7.4) at 4 °C. To purify rhEPO, the dialyzed media was combined with EPO Purification Gel (MAIIA Diagnostics, Uppsala, Sweden), washed, and eluted according to the manufacturer’s directions. Purified rhEPO was then dialyzed against distilled water at 4 °C and stored at −80 °C until use. The concentration was measured using the Quant-iT™ protein assay kit (Invitrogen).

The sialic acid content of the purified rhEPO was measured as previously described^34^. Sialic acid was released from purified rhEPO under mildly acidic conditions. This free sialic acid was then mixed with OPD (*o*-phenylenediamine-2HCl; Sigma) and incubated at 80 °C for 40 min. The amount of OPD-labeled sialic acid was analyzed using a C18 reversed-phase column (Shim-pack CLC-ODS; Shimadzu, Kyoto, Japan) with a fluorescence detector (excitation at 230 nm and emission at 420 nm, Waters).

### N-glycan release and enrichment

N-glycans were released and enriched according to previously optimized procedures^22^. Briefly, purified rhEPO was denatured by rapid thermal cycling (25~100 °C) in a solution containing 100 mM ammonium bicarbonate and 5 mM dithiothreitol. After cooling, the samples were incubated with PNGase F (Promega) at 37 °C for 16 hours. Graphitized carbon cartridges were washed with 80% acetonitrile/ 0.10% trifluoroacetic acid (v/v) in water, followed by conditioning with pure water. The released N-glycan solutions were loaded onto the cartridges and washed with water to remove salts and buffer materials. N-glycans were then eluted by the addition of 40% acetonitrile and 0.05% trifluoroacetic acid (v/v) in water. Finally, the samples were dried under vacuum.

### Profiling the N-glycosylation of rhEPO by LC/MS

Purified N-glycan fractions were separated and analyzed using a nano-LC/Q-TOF MS system. For each sample, 2.0 μL (corresponding to 3 μg of EPO) were injected by an autosampler onto a porous graphitized carbon nano-LC chip (Agilent). A glycan elution gradient was applied to the analytical column at 0.3 μL/min using solutions of (A) 3.0% acetonitrile and 0.5% formic acid (v/v) in water, and (B) 90.0% acetonitrile and 0.5% formic acid in water. N-glycans were eluted with 6% B, 0.00–2.50 min; 6–16% B, 2.50–5.00 min; 16–44% B, 5.00–15.00 min; and 44–100% B, 40.00–50.00 min. The stationary phase was washed out with 100% B for 10 min before returning to 6% B. MS spectra were acquired in positive ion detection over a mass range of m/z 600–2000 with an acquisition time of 1.5 seconds per spectrum. After data acquisition, the raw LC/MS data were processed using the Molecular Feature Extractor algorithm included in the MassHunter Qualitative Analysis software (version B.07.00 SP2, Agilent Technologies). MS peaks were filtered with a signal-to-noise ratio of 5.0 and parsed into individual ion species. Taking into account charge state information, expected isotopic distribution, and retention times, the molecular feature extractor algorithm generated a list of all compound peaks in the sample. Using this information, a list of all compound peaks in the sample was generated, with abundances represented by chromatographic peak areas.

### N-glycan identification by accurate mass

We used in-house computer algorithms to determine N-glycan compositions from LC/MS data by accurate mass^35,36^. Deconvoluted masses were compared against theoretical glycan masses using m/z error tolerance of 5 ppm. According to known glycosylation patterns of recombinant EPOs^23–25^, N glycan compositions consisting of hexose (Hex), N-acetylhexosamine (HexNAc), fucose (Fuc), N-acetylneuraminic acid (NeuAc), O-acetylation (OAc) and dehydration (-H2O) were only considered. High mannose N-glycans or N-glycolylneuraminic acid (NeuGc) were not considered because no associated fragments were examined from LC/MS/MS data.

### Examining the levels of intracellular CMP-Neu5Ac in CHO cells

The intracellular CMP-Neu5Ac concentration of CHO cells was determined as previously described^37^. Briefly, cells were lysed in ice-cold 75% (v/v) ethanol using a sonicator and their supernatants were obtained after centrifugation at 13,000 rpm at 4 °C for 10 min. CMP-Neu5Ac was lyophilized and dissolved in 120 μl of 40 mM phosphate buffer (pH 9.2), followed by centrifugation. Filtrates were obtained using a centricon (MWCO, 10,000) and then added to a CarboPac PA-1 column (Dionex, Sunnyvale, CA). The amount of CMP- Neu5Ac was analyzed using an Abs_260_ absorbance detector (model 486; Waters). The intracellular CMP-Neu5Ac concentration was normalized to the number of cells used in the analysis.

## Electronic supplementary material

Supplementary information
