# Supplementary material for: Inhibition of poly-LacNAc biosynthesis with release of CMP-Neu5Ac feedback inhibition increases the sialylation of recombinant EPO produced in CHO cells
Source: Sci Rep. 2018 May 8;8:7273. doi: 10.1038/s41598-018-25580-9 (PMC5940879; doi:10.1038/s41598-018-25580-9)
Supplement: Supplementary file 1 — Supplementary information [file 41598_2018_25580_MOESM1_ESM.pdf]

Supplementary information

Inhibition of poly-LacNAc biosynthesis with release of CMP-Neu5Ac feedback inhibition increases the sialylation of recombinant EPO produced in CHO cells

Chung-Geun Lee[1†], Myung Jin Oh[2,3†], Seung-Yeol Park[4], Hyun Joo An[2,3] and Jung Hoe Kim[1*]

[1]*Department of Biological Sciences, Korea Advanced Institute of Science and Technology, 291 Daehak-ro, Yuseong-gu, Daejeon 34141, Republic of Korea.*

[2]*Graduate School of Analytical Science & Technology, Chungnam National University, 99 Daehak-ro, Yuseong-gu, Daejeon 34134, Republic of Korea.*

[3]*Asia-pacific Glycomics Reference Site, Daejeon 34134, Republic of Korea*

[4]*Division of Rheumatology, Immunology and Allergy, Brigham and Women's Hospital, and Department of Medicine, Harvard Medical School, Boston, MA 02115 USA*

[†] These authors contributed equally to this work as co-first authors.

[*] To whom correspondence should be addressed:

Jung Hoe Kim

Tel: +82-42-350-2614

Email: kimjh@kaist.ac.kr

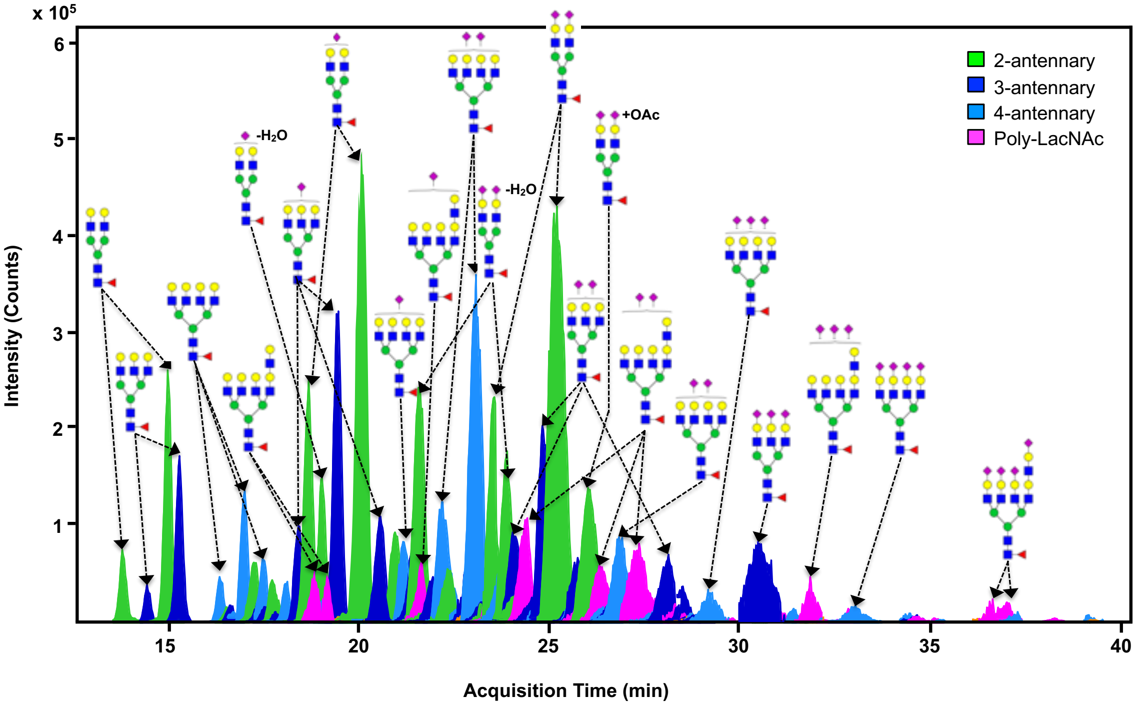

Figure S1. Representative extracted compound chromatograms (ECCs) of N-glycans on rhEPO produced in EC2-1H9 cell by PGC nanoLC-MS. Over 62 individual N-glycan species (including isomers) were detected although not all are visible here due to the wide range of abundances. Color denote different glycan back bone-bi antennary, tri-antennary, tetra antennary, and polylactosamine. Glycan cartoon: green circles:mannose; yellow circles:galactose; blue squares:N-acetylglucosamine; red triangle:fucose; pink diamond: N-acetylneuraminic acid.

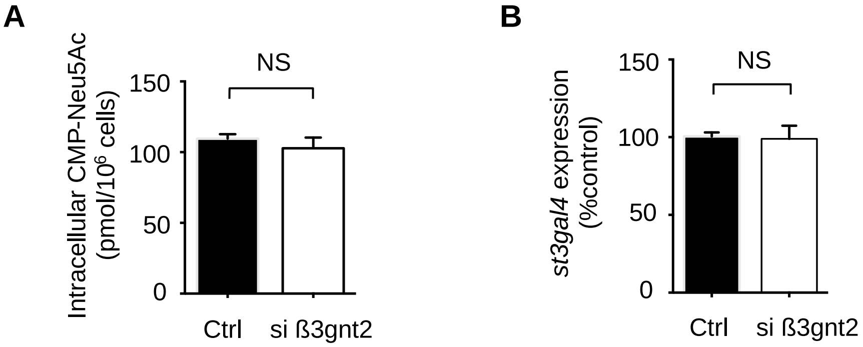

Figure S2. The level of intracellular CMP-Neu5Ac (A) and expression of *st3gal4* (B) was determined in EC2-1H9 cells and β3gnt2-depleted EC2-1H9 cells. β-actin was used as an internal control. Data shown are presented as means ± S.E.M. from three independent experiments each with duplicate samples. NS, not significant (Student's t-test).

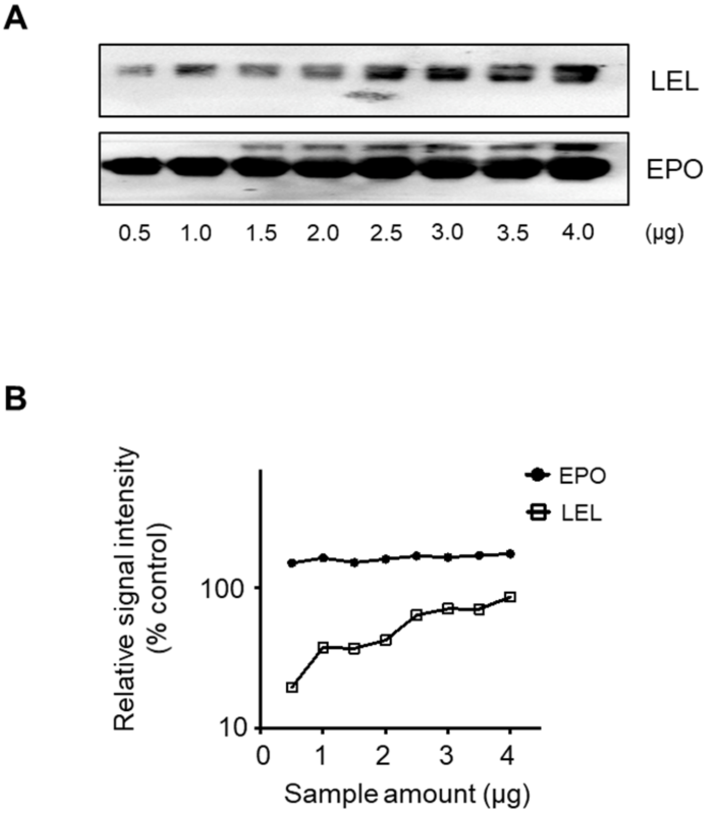

Figure S3. Western blot results with different amount of protein were shown. (A) The signal of LEL (top) and anti-EPO antibody (bottom) was saturated in different sample amount. The signal for polylactosaminylation was in the range of linearity when 1ug of EPO was used. Lane 1 - 8, 0.5 – 4 ug. (B) Quantification of panel A was shown.

Full scan for Fig 2A

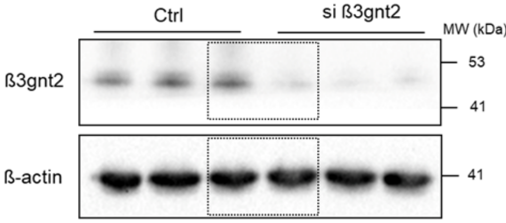

Full scan for Fig 2C

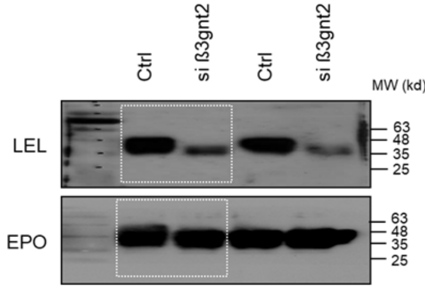

Full scan for Fig 4A

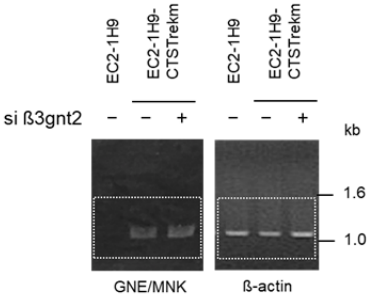

Full scan for Fig 4C

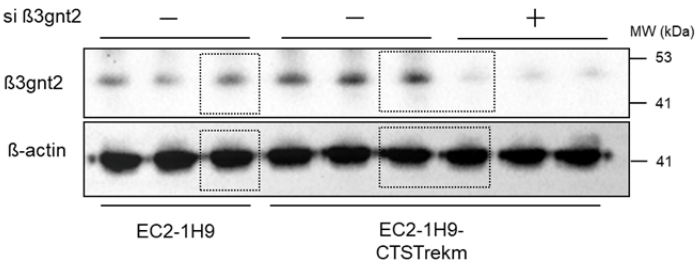

Full scan for Fig 4E

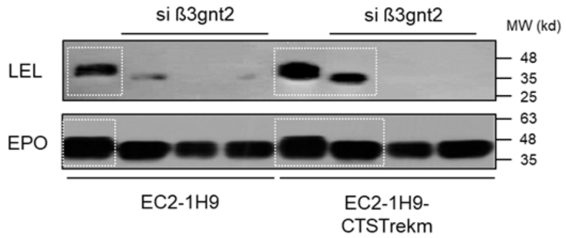

Full scan for Fig S3A

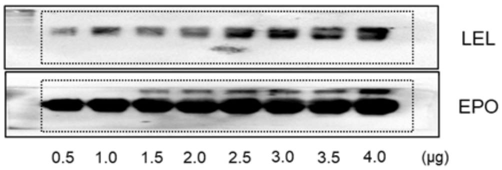

Figure S4. Full scans of gels. Full scans of gels in the indicated figures are shown.

Table S1. Major N-glycan compositions of rhEPO from β3gnt2-depleted EC2-1H9 cells. The table showing the N-glycans with the largest increases in prevalence after treatment with *β3gnt2*-siRNA. N-glycan compositions are presented according to the order of Hexose_N-acetylhexosamine_Fucose_Sialic acid_O-acetylation. Fold increases were calculated as the ratio of the amount of a particular N-glycan in the EC2-1H9 cells before and after β3gnt2-inhibition followed by a normalization against the ratio of total N-glycans between the two samples.

Fold Increase	Relative intensity (%)	N-glycan compositions	Number of branch	Sialic acid capacity	Number of sialic acid
3.07	0.49	$Hex_7HexNAc_6Fuc_1Neu5Ac_1OAc_1$	4	4	1
2.58	3.14	$Hex_7HexNAc_6Fuc_1Neu5Ac_2OAc_1$	4	4	2
1.80	0.68	$Hex_6HexNAc_5Fuc_1Neu5Ac_1OAc_1$	3	3	1
1.74	5.63	$Hex_6HexNAc_5Fuc_1$	3	3	0
1.40	8.69	$Hex_7HexNAc_6Fuc_1Neu5Ac_1$	4	4	1
1.40	1.96	$Hex_5HexNAc_4Fuc_1Neu5Ac_1OAc_1$	2	2	1
1.39	1.85	$Hex_6HexNAc_5Fuc_1Neu5Ac_2OAc_1$	3	3	2
1.29	14.84	$Hex_6HexNAc_5Fuc_1Neu5Ac_1$	3	3	1

Table S2. Relative amounts of N-glycans in each rhEPO samples in figure 5C.

Antennary structure	Relative amounts of N-glycans (%)		
	EC2-1H9	EC2-1H9 CTSTrekm	EC2-1H9 CTSTrekm si ß3gnt2
2-antennary	41.54±2.05	46.61±1.92	38.37±2.70
3-antennary	24.56±1.45	24.94±2.28	35.70±2.06
4-antennary	23.17±1.62	13.35±1.74	26.10±2.05
Poly-LacNAc	10.73±2.12	15.10±1.09	0.83±0.20
